# Potential Effects of Bioactive Compounds of Plant-Based Foods and Medicinal Plants in Chronic Kidney Disease and Dialysis: A Systematic Review

**DOI:** 10.3390/nu16244321

**Published:** 2024-12-14

**Authors:** Esmeralda Josa, Guillermina Barril, Mar Ruperto

**Affiliations:** 1 Department of Nutrition and Bromatology, Universidad Complutense de Madrid, Av. Complutense, s/n, Moncloa—Aravaca, 28040 Madrid, Spain; e.josa.martin@gmail.com; 2 Fundación Investigaciones Biomédicas, C. Pollensa, 2, Las Rozas de Madrid, 28290 Madrid, Spain; g.barril43@gmail.com; 3 Department of Pharmaceutical & Health Sciences, School of Pharmacy, Universidad San Pablo-CEU, CEU Universities, Urbanización Montepríncipe, 28660 Madrid, Spain

**Keywords:** antioxidant, bioactive compounds, chronic kidney disease, inflammation, medicinal plants, plant-based foods

## Abstract

Background. The bioactive components of plant foods and medicinal plants have attracted interest due to their potential impact on the progression of chronic kidney disease (CKD) and outcomes. Objective. This study aimed to conduct a critical and quantitative systematic review of randomized clinical trials (RCTs) investigating the potential effects of selected phytochemicals from plant-based foods and medicinal plants in CKD and dialysis patients. Methods. The review included studies that related plant-based bioactive compounds (curcumin, propolis, sulforaphane, betalain, catechins, rhein, emodin, aloe-emodin, flavonoids, and triptolide) and medicinal plants (green tea, rhubarb, *Astragalus membranaceus*, and *Tripterygium wilfordii* Hook F) in CKD and dialysis patients. A literature search was conducted in PubMed, LILACS, Embase, Scopus, and WOS between December 2022 and October 2024. This review was performed according to the PRISMA flowchart and was registered in PROSPERO (595162). Results. In the eight RCTs conducted with curcumin, anti-inflammatory, antioxidant, and microbiota-modulating properties were reported. As for propolis, in three RCTs, anti-inflammatory, anti-proteinuric, and renal-protective properties were reported. Sulforaphane in one RCT showed antioxidant and cardiovascular benefits, and in another RCT no effects were observed. In one RCT, genistein was shown to be a potential anti-inflammatory agent and improved nutritional status. Allicin in two RCTs showed cardioprotective, antioxidant, anti-inflammatory, and lipid-lowering effects. Finally, beetroot showed a vasodilator effect in one RCT. As for the medicinal plants, green tea, rhubarb, *Astragalus membranaceus*, and *Tripterygium Wilfordii* Hook F, in six RCTs they showed antioxidant, anti-inflammatory, cardioprotective, antiproteinuric, and renoprotective properties. Conclusions. These results suggest that bioactive compounds of plant-based foods and medicinal plants have promising effects in terms of preventing or treating CKD progression and appear to improve inflammation and antioxidant capacity and support cardiovascular benefits and renoprotective effects; however, it is recommended that further studies be carried out.

## 1. Introduction

Chronic kidney disease (CKD) is a public health problem affecting 8.0–16.0% of the world’s adult population. It is expected to rise from the 16th leading cause of death in 2016 to the 5th in 2040 [1,2]. Aging, diabetes mellitus (DM), and cardiovascular disease (CVD) have been identified as the main causes of CKD, with an estimated prevalence of 27.9% to 44.0% in people aged 70 years and older [3]. Consequently, there is growing interest in the search for effective interventions to prevent or slow the progression of CKD and to delay the onset of dialysis. The effects that bioactive components of plant-based foods and medicinal plants may have on the treatment of CKD have attracted much interest because of their potential impact on disease progression and improving patient outcomes.

CKD involves a combination of different pathogenetic mechanisms, such as inflammation, oxidative stress, and endothelial dysfunction, which are closely linked to increased morbidity and mortality [4]. Some inflammatory biomarkers, such as C-reactive protein (CRP), and proinflammatory cytokines, e.g., interleukin-6 (IL-6) and tumor necrosis factor-alpha (TNF-α), can induce oxidative stress through different signaling pathways. On the other hand, oxidative stress also promotes inflammation, establishing a potentially vicious circle through the activation of nuclear transcription factor κB (NF-κB), which is responsible for the recruitment of immune cells. In addition, proinflammatory cytokines associated with oxidative stress play an important role in the pathogenesis of CKD by inducing apoptosis, necrosis, and fibrosis. Uremic toxins also promote inflammation and oxidative stress by priming polymorphonuclear cells, activating interleukin-1 βeta (IL-1β) and IL-8 and the innate immune response [5].

**Figure 1:**
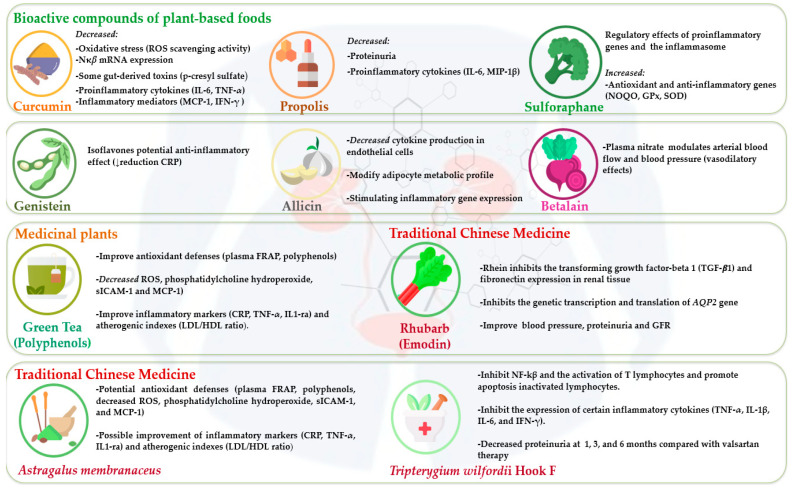
Effects of bioactive compounds from plant-based foods and medicinal plants in chronic kidney disease and dialysis patients.

Bioactive compounds, also termed phytochemicals, are derived from plant-based foods and medicinal plants [6]. Several studies [7,8,9] have suggested that plant-derived compounds, such as curcumin, propolis, sulforaphane, betalain, catechins, rhein, emodin, aloe-emodin, flavonoids, and triptolide, and medicinal plants (rhubarb, *Astragalus membranaceus*, and *Tripterygium wilfordii Hook F*) show potential bioeffects in CKD and dialysis patients. Antioxidant, anti-inflammatory, anticarcinogenic, and renoprotective activity has been attributed to these bioactive compounds [10] (Table 1, Figure 1).

Diet is an important pillar in CKD to delay the progression of the disease and initiation of dialysis. A high fruit and vegetable intake is associated with a reduced risk of developing CKD in the early stages [32]. Despite plant-based protein reducing proteinuria, some authors [26,33] showed that bioactive compounds of plant-based foods and medicinal plants could be involved in the pathogenesis, progression, and complications of CKD. Reducing the current global intake of dietary risk factors could help to reduce CKD-treated end-stage renal disease (ESRD) incidence and the burden of CKD by approximately 3.0% by 2040 [2]. Plant-based diets are rich in protective nutrients and phytochemicals. Among bioactive compounds, some substances actively regulate redox reactions, such as flavonoids, phenolic compounds, carotenoids, anthocyanins, and vitamins C and E [6]. These bioactive compounds interact with reactive oxygen species (ROS) and nitrogen species, contributing to the overall antioxidant defense system in the body and preventing cellular metabolism damage caused by oxidative stress [34]. Dietary antioxidants can act as reducing agents, free radical scavengers, and modulators of enzymatic activity, contributing to redox homeostasis and protecting against oxidative damage.

Furthermore, some antioxidants induce a beneficial response to mild oxidative stress by activating cellular protection and detoxification mechanisms [8,10]. This interaction is complex and depends on multiple factors, including the chemical structure of the antioxidants and their bioavailability.

Recent findings [35,36] demonstrated that the kidney–gut axis plays an important role in the pathogenesis of CKD through a bidirectional interaction between CKD and the host gut microbiota. Accumulating research [35,37] indicates that CKD may influence the composition of the gut microbiota, causing intestinal dysbiosis, which in turn, elevates gut-derived uremic toxins (e.g., indoxyl sulfate), contributing to the progression of CKD, inflammation, and CVD.

Considering that a balanced and personalized diet is essential to maintain nutritional status and improve inflammation and oxidative stress, complementary dietary strategies could be of interest to ameliorate uremic toxicity, improve dysbiosis, delay CKD progression, and improve outcomes in dialysis patients. Therefore, this prompted us to explore the effects of plant-based foods and medicinal plants. Given the particular interest in bioactive compounds, the aim was to conduct a critical and quantitative systematic review of randomized clinical trials investigating the potential effects of selected phytochemicals from plant-based foods and medicinal plants in CKD and dialysis patients.

## 2. Materials and Methods

**Figure 2:**
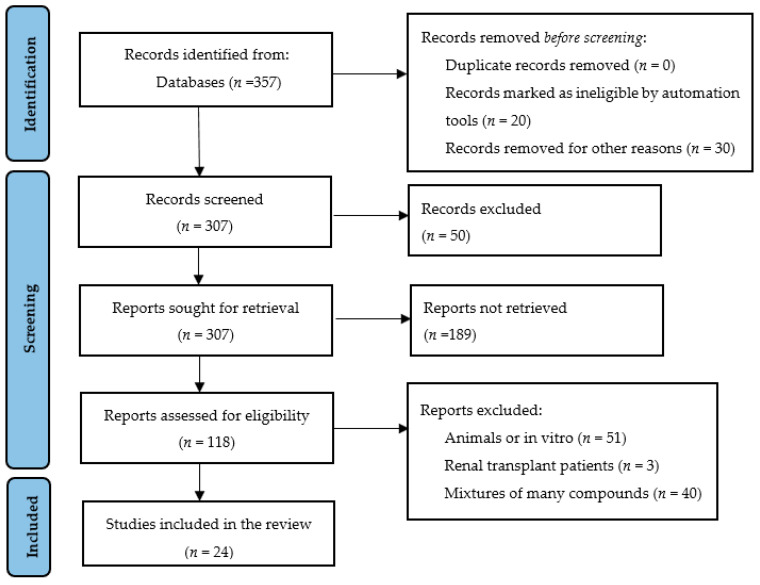
Preferred Reporting Items for Systematic Reviews and Meta-analysis (PRISMA) flow diagram of the systematic review.

This systematic review was conducted following the updated Preferred Reporting Items for Systematic Review and Meta-analysis (PRISMA) (http://prisma-statement.org/prismastatement/Checklist.aspx, accessed on 11 May 2024) guidelines (Figure 2). This review included randomized clinical trials (RCTs) and studies that related plant-based bioactive compounds (curcumin, propolis, sulforaphane, betalain, catechins, rhein, emodin, aloe-emodin, flavonoids, and triptolide), and some medicinal plants (rhubarb, *Astragalus membranaceus*, and *Tripterygium wilfordii* Hook F) in CKD and dialysis patients. A protocol with the main elements of the study was developed and registered with PROSPERO (code number: 595162).

### 2.1. Search Strategy

The search for articles was carried out in the PubMed, LILACS, Embase, Web of Science, and Scopus electronic databases. The following descriptors were used for the database search: “plant bioactive compound”, “phytochemicals”, “diet”, “plant-based”, “herb medicinal”, “medicinal plants”, “chronic kidney disease”, and “dialysis”. In addition, RCTs and studies related to inflammation, oxidative stress, and dysbiosis were considered. These health descriptors were combined with the Boolean operators AND and OR and grouped for sensitivity and specificity. All titles and/or abstracts were assessed, and irrelevant studies were excluded. The articles of interest were then retrieved, and the full texts were reviewed.

### 2.2. Inclusion and Exclusion Criteria

The inclusion criteria included articles published in English and Spanish between December 2020 and October 2024 evaluating human studies with the effects of bioactive compounds obtained from plant-based foods and selected medicinal plants in CKD and dialysis patients. The following exclusion/inclusion criteria were followed using PICO (patient, intervention, comparator, and outcomes): only human subjects with CKD at any stage and dialysis patients were selected, and the full texts of articles without any time filters were considered suitable for inclusion. The following exclusion and inclusion criteria were adopted for the full-text articles: RCT and quasi-RCT clinical trials with a mean duration of ≥ 4 weeks were included; interventions targeting the consumption of animal-derived bioactive nutrients, vitamins, minerals, toxic compounds, or other nutritional oral supplements were excluded; and interventions analyzing the effects of single isolated bioactive compounds (curcumin, etc.) were included. Any diet or study demonstrating a lack of adherence or compliance in the pre-post study was excluded. Review articles, theses, abstracts in scientific congress, letters to the editor, case reports, animal or in vitro studies, and studies on renal transplant patients and mixtures of many compounds were excluded. Articles repeated in different databases and articles that duplicated information from a previously included population were discarded. After duplicates were removed, the titles and abstracts from all reports were screened, full texts were independently examined for eligibility, and data were extracted by the authors individually. A meta-analysis was not applicable because of the heterogeneity of the studies, approaches, and results.

### 2.3. Study Selection 

The research simultaneously and independently identified and selected the articles in all the databases searched between January 2000 and October 2024. Disagreements between the reviewers were resolved by consensus. In this stage, there were no language or period restrictions for choosing the publications. The studies were selected by evaluating the titles, reading the abstracts, and subsequently reading the full texts of the studies, observing the inclusion and exclusion criteria. Additionally, the reference lists of the chosen articles were analyzed to identify any study that had not been captured using the search strategy. The Critical Appraisal Skills Programme (CASP) Systematic Review Checklist (https://casp-uk.net/casp-tools-checklists/systematic-review-checklist/, accessed on 12 May 2024) was used to assess the reliability, relevance, and results of articles included in this systematic review.

Figure 2 shows the PRISMA flow diagram of the systematic review of plant-based foods and medicinal plants. In the first search, 357 articles were identified in the databases. Of these, 50 were initially discarded after reading the titles. On further screening, which consisted of reading and analyzing the abstracts, 189 articles were excluded, of which 118 were assessed for eligibility for full-text reading according to the PICO strategy. After this evaluation, 23 RCTs and 1 clinical trial were included in this systematic review. The results were described in two parts: plant-based bioactive compounds (*n* = 18 articles) and medicinal plants (*n* = 6 articles). The results of the studies are summarized in 

### 2.4. Data Extraction

Data were extracted independently by 3 researchers (E.J., G.B., and M.R.) and included in an Excel spreadsheet containing the following items: authors, year of publication, bioactive compound, study design, type and number of patients (CKD 1–5, hemodialysis or peritoneal dialysis), dosage, molecular and therapeutic effects, and major findings associated with bioactive-compound interventions. The information extracted from the articles was used to build an explanatory model regarding the factors associated with plant-based foods and medicinal plants and their potential effects in CKD and dialysis patients.

### 2.5. Evaluation of the Quality of the Articles

The Jadad scale [38] was used to assess independently the methodological quality of the RCTs included in this systematic review. For each item of the study analyzed, a response and a code were attributed: yes (1) or no (0), with the score of the questionnaire ranging from 0 to 5 points. An RCT was of high methodological quality, assuming a low risk of bias if the Jadad score was ≥ 3 points. In the methodological quality assessment of the 23 included RCTs, 4 articles had a Jadad score of 4 points (21.05%) [39,40,41,42] and 19 articles (78.95%) met all the checklist criteria [12,13,14,43,44,45,46,47,48,49,50,51,52,53,54,55,56,57,58]. Studies with Jadad scale scores < 3 points were excluded due to their low quality and high risk of methodological bias. The quality classification was carried out independently by 3 researchers (E.J., G.B., and M.R), and any differences were resolved through discussion.

## 3. Results

### 3.1. Potential Effects of Plant-Based Food Bioactive Compounds on Chronic Kidney Disease

#### 3.1.1. Curcumin

Curcumin, found in turmeric (Curcuma Longa), is a phenolic compound with a variety of biological activities in CKD. It is a yellow-colored spice that comes from a flowering plant of the ginger family and is native to Asia and Central America. Previous studies [11,12,13,14] have reported the bioeffects of curcumin on inflammation, oxidative stress, and CKD progression. Table 2 shows the clinical trials of curcumin and its therapeutic effects in CKD and dialysis patients. 

Inflammation per se accounts for 30–50% of CKD patients, contributing significantly to CKD progression and poorer prognosis in dialysis patients [59]. Causative factors, such as oxidative stress and cellular senescence, hypoxia, fluid and sodium overload, immune dysfunction, and intestinal dysbiosis, as well as accumulation of uremic toxins that emerge from molecular and metabolic derangements in the uremic milieu, were reported [60,61]. 

The effects of curcumin in CKD and dialysis patients have been related to molecular mechanisms such as scavenging superoxide radicals, hydrogen peroxide, and nitric oxide (NO). All of these molecular mechanisms were associated with the amelioration of oxidative stress through increased expression of antioxidant enzymes, activation of nuclear factor erythroid factor 2 (Nrf2), scavenging of ROS, and downregulation of inflammatory cytokines (IL-6 and TNF-α) modulated by nuclear factor κappa β (NF-kβ) [12,13,14]. Two double-blind randomized clinical trials [43,44] demonstrated that curcumin was an effective adjuvant therapy for oxidative and inflammation modulation in hemodialysis (HD) patients. One hundred and nineteen HD patients were either treated with 500 mg turmeric (4.42% curcumin; one capsule/three times per day) or a placebo for three months of follow-up. In the turmeric-supplemented group, a significant reduction in plasma IL-6 and TNF-α concentrations was found compared with the placebo group. In this study, turmeric supplementation in HD patients was safe and effective as an anti-inflammatory agent. Another RCT carried out on 28 HD patients [39] analyzed the expression of inflammatory transcription factors, such as NF-kβ, Nrf2, Nod-like receptor pyrin domain containing-3 (NLRP3), and IL-1β. The intervention group received after HD a juice composed of 100 mL orange juice, 12 g carrot, and 2.5 g turmeric (95% curcumin) administered three times per week. Interestingly, the findings suggested that curcumin was an effective anti-inflammatory supplement by decreasing CRP levels, NF-kβ, and mRNA expression in patients who received HD. 

Oxidative stress is a risk factor for morbidity and mortality in CKD and dialysis patients [62]. The mechanisms of action of the kidney’s antioxidant barrier can slow down oxidative stress under physiological conditions. Oxidative stress has been linked to the production of highly reactive intermediate molecules in the presence of inflammation, such as superoxide anion (O2-) and hydroxyl (OH-) free radicals. The hyperproduction of ROS can further enhance the inflammatory response by activating pro-inflammatory mediators through the NF-κβ metabolic pathway [63]. 

Curcumin, as a phenolic antioxidant compound, has also been studied as an adjuvant therapy to prevent or attenuate oxidative stress and as an anti-inflammatory agent in non-diabetic and diabetic CKD patients. In a randomized double-blind placebo-controlled clinical trial [14], the role of curcumin supplementation was studied in the profiles of redox status and Nrf2 activation in non-diabetic proteinuric CKD patients compared with diabetic proteinuric CKD patients. Fifty-two volunteers were supplemented with 107 mg of curcumin in each meal (320 mg/day) or placebo for 8 weeks. The curcumin-supplemented group showed improved free radical scavenging activity and attenuated serum levels of malondialdehyde (MDA)—an oxidative stress marker in non-diabetic proteinuric CKD patients. Likewise, a double-blind, placebo-controlled RCT carried out in HD patients [12] studied the potential antioxidant and anti-inflammatory response to curcumin supplementation. Forty-three HD patients were randomized into two groups to receive curcuminoid capsules (1 g/day) versus placebo for 12 weeks. As oxidative stress markers, catalase (CAT), glutathione peroxidase (GPx), glutathione reductase (GR) activity, and MDA, together with high-sensitivity CRP, were analyzed. The curcuminoid intervention showed a marked increase in CAT activity, whereas no changes were observed in GPx, GR, MDA, and CRP concentrations.

The plausible explanation for the above results was associated with the heterogeneity of the population analyzed (CKD, dialysis), sample size, the duration of the RCTs, and the dosage of curcumin used. 

A CKD stage 3–4 double-blind placebo-controlled clinical trial [40] examined if curcumin supplementation had anti-inflammatory and antioxidant effects in the prevention of contrast-induced nephropathy (CIN) following coronary angiography or angioplasty. Thirty CKD patients in stages 3–4 were selected to be supplemented with curcumin, and thirty were given a placebo. Curcumin supplementation (500 mg/3 times a day) was administered from 2 days before the intervention until 3 days post-procedure. Serum creatinine and urinary neutrophil gelatinase-associated lipocalin were analyzed before the procedure and on days 2 and 3 after angiography. No significant results were obtained with curcumin in ameliorating the possible adverse effects of CIN in patients with stage 3–4 CKD.

Based on the findings of previous studies [12,14,40], curcumin supplementation could be considered a complementary dietary strategy to prevent or ameliorate oxidative stress and inflammatory response in CKD and dialysis. On the other hand, the protective effect of curcumin in reducing the effect of CIN in patients with stage 3–4 CKD remains unclear. Further randomized clinical trials with large sample sizes are needed to determine the dosage of curcumin to attenuate inflammation and reverse oxidative stress in CKD and dialysis patients.

Emerging evidence suggests that gut microbiota dysbiosis is involved in CKD progression and plays a key role in the gut–kidney axis. Therefore, increased attention has been given to gut microbiota as a novel approach to treating CKD. A placebo-controlled double-blind clinical trial [45] was conducted on twenty-four stage 3a–4 CKD patients. The study aimed to evaluate the effect of curcumin supplementation on inflammation, lipid peroxidation, and gut microbiota diversity. The curcumin-supplemented therapeutic arm was given 500 mg of curcumin/twice a day for 6 months compared to placebo (controls). A significant reduction in pro-inflammatory mediators [monocyte chemoattractant protein-1 (MCP-1 or CCL-2), interferon-gamma (IFN-γ), and interleukin-4 (IL-4)] and lipid peroxidation markers (thiobarbituric acid reactive substances (TBARS)) were found to be associated only with curcumin intervention. No significant changes were observed in the placebo group. After 6 months of curcumin supplementation, gut alpha diversity was observed to be similar to that of healthy subjects. At the phylum level, gut diversity was more balanced at the expense of a significant increase in *Lachnoclostridium* spp. and a decrease in *Escherichia* spp. and *Shigella* spp. It is also noteworthy that, at the family level, *Lactobacillaceae* spp. increased significantly in the last 3 months of curcumin supplementation. 

Several potential mechanisms may explain the relationship between gut microbiota diversity and inflammation in CKD. Firstly, gut microbiota imbalance directly influences inflammatory cytokine production, contributing to a progressive decline in renal function and characterized by a gradual accumulation of gut uremic toxins [indoxyl sulfate (IS) and p-cresyl sulfate (pCS)], resulting from abnormal gut microbial metabolism. Additionally, gut microbiota can influence the host immune system. The gut is the largest immune organ, and the gut microbiota plays an important role in immune maturation, homeostasis, and tolerance. Changes in gut microbiota can lead to intestinal inflammation and increased intestinal barrier permeability, contributing to the activation of the immune system. Second, CKD is also associated with changes in the composition of the gut microbiota, and reduced gut microbiota diversity may affect interactions between taxa, leading to an inflammatory response. Third, gut microbiota can regulate the production of indole and indole derivatives, such as indoleacetic acid and tryptamine (TMA) from tryptophan, promoting gut homeostasis, preventing inflammatory responses, and inhibiting CD4+ T cell and B cell activation. Gut microbiota can also produce TMA from choline and carnitine and then convert it to trimethylamine-N-oxide (TMAO) in the liver. This is considered a novel modulator of inflammation, and microbial metabolism of TMAO is associated with the activation of pro-inflammatory factors and the development of various inflammatory conditions. CKD results in very high production of TMAO, which directly influences the development of vascular calcification and atherosclerosis [35,36]. Several gut-derived uremic toxins, such as IS, pCS, indole-3-acetic acid (IAA), TMAO, and phenylacetylglutamine, have been closely linked to inflammation, vascular calcification, CVD, and dysbiosis in CKD and dialysis patients [35,37,64]. A randomized double-blind placebo-controlled clinical trial carried out in HD patients [13] evaluated the effect of curcumin supplementation compared to placebo on reducing plasma levels of gut uremic toxins. Fourteen patients were randomly assigned to the curcumin group and received 100 mL of orange juice and 2.5 g of turmeric, and 14 controls received the orange juice without turmeric three times per week after the HD session. After three months of follow-up, the curcumin-supplemented group exhibited significantly reduced pCS, whereas no differences in IS or IAA were found in either group. The study results suggested that curcumin supplementation three times a week post-HD session helps to mitigate inflammation and oxidative stress. The gut microbiota is a complex ecosystem, and its metabolites can modulate host metabolism, immunity, and pathophysiology by influencing signaling pathways, genes, proteins, and maintenance of gut barrier integrity in CKD and dialysis patients. Increased intestinal permeability has been related to hyperammonemia ions in the intestinal lumen, alteration of tight junction proteins (claudin-1, occludin, and tight junction ZO1 protein) in the colonic mucosa, and translocation of bacterial products of intestinal origin, as shown by the presence of DNA fragments of aerobic and anaerobic pathogens of intestinal origin circulating in patients with CKD and on dialysis [36,65]. An increase in circulating gut-derived bacterial products activates innate immunity and inflammatory cell recruitment, which results in increased production of circulating proinflammatory cytokines. Thus, the interaction between tight junction disruption and inflammation creates a vicious cycle that potentiates intestinal and systemic inflammation and intestinal barrier dysfunction [66].

In the eight RCTs included in this systematic review, curcumin or turmeric supplementation showed no adverse effects in CKD or dialysis patients. The RCTs used doses of curcumin between 367 and 1500 mg/day, while the doses of turmeric supplements were between 500 and 2500 mg/day.

Turmeric longa, with its bioactive compound curcumin, given its low cost and lack of side effects in RCTs, was shown to be a potential anti-inflammatory and antioxidant supplement. Moreover, it was shown to reduce the production of gut-derived uremic toxins through modulation of the gut microbiota in CKD and HD patients. Various studies [67,68] have recommended mixing curcumin powder with black pepper, due to the bioactive compound piperine, which has been shown to enhance absorption and bioavailability. More RCTs are needed to investigate the molecular mechanisms, metabolic pathways, therapeutic dosage, and clinical effects of curcumin in CKD and dialysis patients.

#### 3.1.2. Propolis

Propolis is a natural resin-like substance collected by honeybees from various plant sources, including buds and exudates, and is mixed with wax, honey, and salivary secretions [69]. Over 300 bioactive compounds have been identified in propolis, mainly related to phenolic compounds such as flavonoids, terpenes, aromatic aldehydes, beta-steroids, and alcohols [15]. The composition of propolis varies with geographical location, plant source, season, productivity, bee species, and harvesting method [16]. Propolis has been utilized in studies to evaluate biological properties, such as anti-inflammatory, antioxidant, antifungal, antitumoral, and immunomodulatory activities [15,16,17]. Table 3 shows the RCTs conducted with propolis in CKD and dialysis patients. 

Some RCTs [41,70,71], have reported effects of propolis on CKD or dialysis patients and its nephroprotective properties and/or effects on inflammatory response and oxidative damage. The molecular mechanisms of propolis involved blocking NF-κβ localization, inhibiting ROS, regulating gene expression, and reducing oxidative stress and inflammation by modulating the TNF-α pathway [72]. The anti-inflammatory effects of propolis are mediated by its inhibition of the expression of Toll-like receptors 2 and 4. Also, propolis contains flavonoids such as kaempferol, galangin, pinocembrin, acacetin, isorhamnetin, aurapten, and 5,7-dimethoxy-8-flavonol that have potent antioxidant effects [73].

The effect of green propolis has been a matter of interest in CKD. A randomized placebo-controlled clinical trial in thirty-two CKD stage 2–4 patients [41] analyzed the impact of Brazilian green propolis extract on proteinuria and changes in glomerular filtration rate (GFR). Measurements were performed for albuminuria, proteinuria, changes in GFR, and the urinary level ofMCP-1 as an inflammatory marker. Participants were randomized to receive 500 mg/day of green propolis (intervention group) or placebo (controls) for 12 months follow-up. In the propolis-supplemented group, proteinuria was significantly improved (695 mg/day vs. 1403 mg/day) and there was a significant reduction in urinary excretion of MCP-1 (58 pg/per mg of creatinine vs. 98 pg/per mg of creatinine) post-intervention compared to the controls. Furthermore, the hepatic, muscle, and pancreatic blood markers analyzed in the study showed no significant changes during the 12-month follow-up. This RCT demonstrated that propolis reduced proteinuria and inflammatory status with no adverse effects associated with supplementation in CKD stage 3b patients.

Due to the multifactorial nature of the inflammatory process in CKD, a multimodal approach with propolis could be of interest. Inflammatory markers, such as serum CRP, are higher in dialysis patients than in the general population, which may be a result of increased cytokines IL-1β, IL-6, and TNF-α [60]. A prospective open-label clinical trial [70] evaluated the safety of the inflammatory status of green propolis extract in 37 HD patients. Patients were allocated to receive 200 mg/day of dehydrated Brazilian green propolis extract (EPP-AF^®^) for 4 weeks followed by a washout period without propolis. Plasma levels of IFN-γ, selected cytokines (IL-1β, IL-1α, IL1RA, IL-4, IL-6, IL-8, IL-10, IL-12, IL-13, IL-17, and TNF-α), and serum CRP were measured as the primary endpoints for the assessment of inflammatory parameters. To evaluate the potential for hepatic, pancreatic, or muscle toxicity, analyses were performed before and after the use of EPP-AF^®^ with AST, ALT, pancreatic amylase, and CPK. The results from the study showed that a significant reduction in IL-6, TNF-α, INF-γ, IL1Ra, IL-17, IL-13, and IL-8 was found after propolis supplementation. Notably, in this clinical trial, EPP-AF^®^ supplementation was shown to be safe and associated with a significant and sustainable reduction in proinflammatory cytokines after a 4-week post-trial period. Therefore, the above findings show the immunomodulatory activity of EPP-AF^®^ in HD patients. Additionally, a double-blind controlled clinical trial in 41 HD patients [46] was randomized to EPP-AF^®^ green propolis dry extract (400 mg/day) or placebo for two-month follow-up. The effect of propolis on plasma TNF-α and macrophage inflammatory protein-1β (MIP-1β) levels was examined. The propolis-supplemented group exhibited significantly reduced concentrations of TNF-α, with a non-significant trend of reduced MIP-1β levels. No substantial changes were observed in the placebo group. 

In the aforementioned studies [41,46,70], the results demonstrated the anti-inflammatory and long-lasting effect of supplementation with dehydrated green propolis extract at different dosages in CKD and HD patients. Similarly, the effects of propolis were studied in peritoneal dialysis (PD). A randomized clinical study [47] in 22 PD patients assessed the inflammatory effect of propolis. PD patients were randomly allocated to receive a concentrated standardized dry extract of green propolis (400 mg/day) or a placebo for two months. Plasma levels of proinflammatory cytokines TNF-α and IL-6 were measured, and quantitative polymerase chain reaction analyses were performed to evaluate the transcriptional expression levels of Nrf2 and NF-κB in peripheral blood mononuclear cells (PBMCs). Plasma MDA levels, a lipid peroxidation marker, as well as serum CRP were analyzed. The results showed a significant reduction in TNF-α (*p* = 0.02) after propolis intervention, but no statistical changes in MDA, IL-6, or CRP concentrations were found. A non-significant trend of fold-change increases in Nrf2 expression after two months of intervention was observed in the propolis group. The supplementation of EPP-AF^®^ green propolis extract (400 mg/day) for two months ameliorated inflammation, decreasing the TNF-α plasma concentrations in PD patients. Further RCTs are required to explore the effect of propolis supplementation on inflammatory and oxidative markers in CKD and dialysis populations.

Propolis, as a bioactive compound, can be proposed for use as a natural adjuvant for its possible nephroprotective and anti-inflammatory and antioxidant effects in CKD and dialysis patients. So far, RCTs conducted with propolis in CKD and dialysis have shown no remarkable adverse effects of green propolis extract supplementation with dosages between 400 and 500 mg/day. Given the limited number of conducted trials at present, more long-term randomized clinical trials are needed to investigate the properties of propolis in the different stages of CKD and dialysis modalities.

#### 3.1.3. Sulforaphane

Sulforaphane is an isothiocyanate naturally found in cruciferous vegetables (Brassicaceae), such as broccoli, cauliflower, cabbage, kale, brussels sprouts, kohlrabi, and collard greens [74]. Hydrolysis of cruciferous vegetables such as broccoli and Brussels sprouts yields immunomodulatory isothiocyanates, most notably sulforaphane (1-isothiocyanato-4-methylsulfinylbutane). Studies [18,74] have shown that sulforaphane-based interventions may represent a novel therapeutic approach in CKD and dialysis patients (Table 1 and Table 3).

Sulforaphane is a potent antioxidant and pro-oxidant with the ability to promote endogenous antioxidants as a natural activator of the Nrf2/Keap1 cytoprotective pathway, considered a master regulator of cellular antioxidant responses [18]. It reduces the accumulation of ROS; restores the renal activities of superoxide dismutase-1, CAT, and GPx; and enhances intracellular glutathione in high-glucose-treated renal glomerular mesangial cells and tubule cells in vitro [18]. There is no acceptable daily intake (ADI) or medium daily intake (MDI), but at least three servings of about 150–200 g per week of cruciferous vegetables are recommended [75]. Recent studies [18,19,20] have demonstrated that sulforaphane has numerous biological properties as an antimicrobial, antioxidant, anti-inflammatory, and anti-oncogenic agent and as an epigenetic modulator. Sulforaphane has been reported to act as an antibacterial agent against Helicobacter pylori by inhibiting bacterial urease synthesis [20], and it has also been reported that Nrf2 activators have a potential role in the treatment of COVID-19 [76] viral pneumonia via benefiting the pulmonary antibacterial defense system. Consequently, there is great interest in sulforaphane as a bioactive compound in CKD. A recent randomized double-blind crossover study in 30 HD patients [42] aimed to evaluate the effects of sulforaphane on the expression of Nrf2 and NF-κβ. Fourteen HD patients were randomly assigned to the sulforaphane intervention (one sachet/day of 2.5 g containing 1% extract and 0.5% myrosinase) and sixteen patients to the placebo group (one sachet/day of 2.5 g containing corn starch colored with chlorophyll) for 2-month follow-up. After the 2-month washout period, the groups were switched. Nrf2 and NF-κβ mRNA expression levels were assessed by polymerase chain reaction as inflammatory parameters and serum MDA was assessed as a marker of lipid peroxidation. The sulforaphane supplementation (150 μmol/day) showed no significant changes in Nrf2 or NF-kβ mRNA expression. Likewise, no significant differences were observed in proinflammatory cytokines (IL-6 and TNF-α) or MDA levels. No adverse effects were found with the sulforaphane extract intervention. The authors pointed out that 150 μmol/day had no anti-inflammatory or antioxidant effect on HD patients. Therefore, the biological properties of sulforaphane remain unclear, and further RCTs are needed to elucidate the efficacy of sulforaphane supplementation in HD patients.

However, another recent randomized placebo-controlled clinical trial in twenty-five CKD stage 3–5 patients [48] evaluated the effects of sulforaphane on the mRNA expression of Nrf2, NF-κβ, and markers of oxidative stress (lipid peroxidation and protein carbonylation). Patients in the sulforaphane group were given 400 μg of L-sulforaphane daily (60 capsules of 200 μg). For one month, including weekends, they were instructed to take two capsules daily and to do so between meals to prevent potential interactions with other nutrients. The placebo group received 400 μg of cornstarch daily. The sulforaphane group showed a significant increase in the mRNA expression of Nrf2 and quinone oxidoreductase 1 (NQO1), as well as improvements in phosphate, glucose, and triglyceride levels. In contrast, the placebo group experienced an increase in plasma levels of low-density lipoproteins (LDLs) and total cholesterol, highlighting the positive effects of sulforaphane on reducing oxidative stress, as well as its ability to improve cardiovascular parameters.

The findings from this systematic review suggest that it is important to standardize the sulforaphane dosage and treatment duration in CKD and dialysis patients in future studies. Although a few non-RCTs have used this compound as a supplement in CKD or dialysis patients, the safety profile of sulforaphane supplementation was considered favorable, with no statistically significant differences in adverse effects between treatment and placebo groups. Based on this background, future interventions with standardized doses of sulforaphane should be tested in longer-term clinical trials to establish its potential as a modulator of inflammatory and oxidative stress in CKD, as well as for delaying the onset of dialysis.

#### 3.1.4. Genistein

Genistein is a small, hydrophobic molecule with a molecular weight of 270 Daltons. It is an isoflavone considered to be a phytoestrogen. Genistein is mostly found in leguminous plants such as soybeans (Glycine max), chickpeas (Cicer arietinum), and broad beans (Vicia faba), which contain approximately 11–35 mg of isoflavones in 100 g of dry beans. The bioavailability of genistein ranges from 0.1 to 4.0%. Exogenous genistein is absorbed in the digestive tract, metabolized, and distributed by attaching itself directly to plasma proteins. More than 30 aromatic hydroxylation and demethylation metabolites of genistein have been detected in urine, plasma, and human tissues. Among the soybean isoflavones, genistein is considered the most predominant in the human diet [21]. To date, the ADI and MDI have not been established. Genistein supplementation has shown promise in the management of CKD. Some studies [21,22] have indicated that genistein supplementation may help regulate blood pressure by affecting renin secretion and inhibiting cellular apoptosis through inhibition of SIRT1 gene expression in common kidney diseases (diabetic nephropathy, hypertensive kidney disease, and renal fibrosis). 

Additionally, genistein supplementation has demonstrated effects on calcium and phosphate balance, with decreased urinary calcium content and increased serum vitamin D. Furthermore, genistein has been found to have diuretic effects by reducing vascular resistance and protecting the permeability barrier of the kidney, inhibiting the increase in albumin permeability in acute glomerular inflammatory injury. These findings highlight the multifaceted potential benefits of genistein supplementation in CKD patients [22]. Table 1 and Table 4 show the biological effects of genistein in CKD and dialysis patients.

Research has suggested that genistein may play a role in modulating the inflammatory response and oxidative stress [22]. A randomized double-blind clinical trial study [49] hypothesized that dietary soy isoflavones improved systemic inflammation in inflamed HD patients. Inflammation was measured by CRP, IL-6, and TNF-α, and nutrition parameters were studied by serum albumin, prealbumin, insulin-like growth factor-1 (IGF-1), and normalized protein catabolic rate (nPCR). Thirty-two inflamed HD adults were administered soy-based nutritional supplements with isoflavones (54 mg in a protein drink during the scheduled dialysis session or 26 mg in a protein bar or something similar on non-dialysis days) (soy group) vs. milk protein without isoflavones (control group) for 8 weeks. The supplemented group showed a positive correlation of total isoflavones and genistein with serum albumin and IGF-1 and an inverse association of total isoflavones with CRP level. Previous findings supported an anti-inflammatory, nutritional effect of dietary soy in HD patients with underlying systemic inflammation. Specifically, the proposed molecular mechanisms are related to an enhancement of the inflammatory response through the downregulation of several inflammatory factors [IL-1, IL-6, NF-κβ, TNF-α, Toll-like receptor 4, and serum CRP]. Of note, genistein also inhibits oxidative stress (protein carbonyl, MDA, and Nrf2), which protects against oxidation of DNA, proteins, and lipids. Moreover, it is involved in angiogenesis through the inhibition of vascular endothelial growth factor (VEGF) and fibroblast growth factor (FGF) in kidney cancer [22].

Also, there is some experimental evidence to suggest that the progression of CKD is slower given diets based on soy protein. A crossover clinical trial [23] compared the effect of a soy-based vegetarian protein diet (VPD) and an animal-based low-protein diet (APD) in CKD stages 3–4. The patients were randomly assigned to either group 1 (VPD) or group 2 (APD). They stayed on one diet for 6 months and then switched to the other diet for a second 6-month period. The VPD was well tolerated; decreased 24 h urinary creatinine and phosphate and serum CRP; and maintained GFR. These potential advantages may have protective effects, specifically in CKD stages 3–4.

Genistein, as an isoflavone and soy derivative, may be useful in slowing the progression of CKD and as an anti-inflammatory agent in patients with CKD, such as those on dialysis. Potential drug interactions are a crucial consideration when incorporating genistein supplementation into the treatment of CKD and dialysis patients. Genistein’s impact on the renin–angiotensin–aldosterone system and blood pressure regulation suggest caution when combined with antihypertensive angiotensin-converting enzyme (ACE) inhibitors targeting similar physiological pathways. The potential use of genistein as a diuretic warrants more in-depth clinical research, particularly to assess its efficacy and safety in CKD and dialysis patients. Additionally, its influence on calcium and phosphate balance may interact with drug targeting. Furthermore, the varying effects of genistein on kidney permeability barriers and tight junctions highlight the complexity of its interactions with medications affecting kidney function, necessitating careful monitoring and assessment of potential drug combinations. 

#### 3.1.5. Allicin

Allicin, a sulfur-containing compound, is synthesized from alliin through the action of the enzyme alliinase. The chemical structure of allicin consists of two prop-2-ene-1-sulfinothioic acid S-allyl ester molecules linked by a disulfide bond, giving it its characteristic odor. It is primarily found in garlic (*Allium sativum*) and other related Allium species, such as ramsons (*Allium ursinum*) and hooker chives (*Allium hookeri*). These plants produce allicin as a defense mechanism when their cells are damaged, and it has been reported to be one of the primary substances responsible for garlic’s antiviral activity and immunomodulatory, anti-inflammatory, and antioxidant effects [26]. It is important to note that allicin is particularly active against several multidrug-resistant strains of human pathogens, including methicillin-resistant *Staphylococcus aureus* and *Helicobacter pylori*.

The natural sources of allicin, particularly garlic, have long been recognized for their potential health benefits. Allicin’s ability to promote the production of antioxidant enzymes and inhibit the oxidation of LDL particles underscores its potential in ameliorating oxidative stress-related complications often observed in CKD patients. Allicin is considered a superoxide anion radical scavenger that can ameliorate lipid peroxidation. Moreover, it can activate Nrf2, which is responsible for the activation of antioxidant response elements and the production of phase II antioxidant and detoxifying enzymes, such as glutathione (GSH), SOD, CAT, and GPx [77]. Table 4 shows the RCTs of allicin and its bioeffects in CKD and dialysis patients. 

Asgharpour et al. [50], in a randomized double-blind clinical trial, evaluated the potential use of garlic extract to improve lipid profiles, reduce inflammation, and modify cardiovascular (CV) risk factors. Seventy HD patients were randomized to receive 300 mg of standardized garlic powder (containing 1.3 milligrams of garlic extract) or a placebo for eight weeks. After a six-week washout period, the two groups were switched so that the group that received garlic powder in the first eight weeks received a placebo for the second eight weeks and vice versa. In this study, garlic extract was useful in reducing triacylglyceride, oxidized LDL, and homocysteine levels. These findings underscore the potential of allicin, as a component of garlic extract, to address the critical aspects of managing CKD and its associated complications, particularly in the context of oxidative stress and inflammation. A further randomized double-blind clinical trial [51] evaluated the effect of garlic extract on the inflammatory markers (IL-6, CRP, and erythrocyte sedimentation rate) of 40 patients undergoing PD. The patients received a dosage of 400 mg of a standardized garlic extract, including 1 mg of alliin or a placebo twice a day for 8 weeks. The garlic-supplemented group showed significantly reduced IL-6, CRP, and erythrocyte sedimentation rates, while in the placebo group a significant decrease was only observed in plasma IL-6. These properties suggest that allicin may hold the potential for mitigating oxidative stress and inflammation, as well as potentially addressing bacterial complications that can arise in dialysis patients. Therefore, the multifaceted properties of allicin present a compelling rationale for further investigation into its potential benefits for individuals with CKD, particularly in the framework of oxidative stress, inflammation, and potential antimicrobial effects.

#### 3.1.6. Betalain from Beetroot (*Beta Vulgaris Rubra*)

The tuber *Beta vulgaris rubra*, also known as beet, has aroused great interest as a functional food for its impact on human health [24]. Beetroot is a source of inorganic nitrate, which can be reduced to form nitrite and nitric oxide (NO) after dietary intake. NO is an important endogenous signaling molecule in biological systems and has been implicated in a wide variety of physiological and pathological processes, including blood pressure regulation, immune defense, and neurotransmission. NO was first identified as an endothelial-derived relaxing factor and has well-established vasodilatory properties, playing essential roles in the regulation of renal blood flow, GFR, and blood pressure. It is increasingly being recognized that the reduced bioavailability of NO due to decreased production and/or increased scavenging contributes to the pathogenesis of CVD, including hypertension, atherosclerosis, myocardial ischemia and infarction, and heart failure. It has recently been recognized that the intake of nitrates from vegetables is an important exogenous source of NO with vasodilator activities [24,25,78].

However, excessive or dysregulated NO production can lead to harmful effects. In pathological conditions such as infection, an overproduction of NO may contribute to tissue damage and worsen inflammatory responses. NO can interact with ROS to produce reactive nitrogen species, such as peroxynitrite, which can induce oxidative and nitrosative stress, causing cellular damage and playing a role in the development of various diseases, including CVD [25]. Production of NO in the body is both a positive and negative process, with its effects being highly context-dependent.

*Beta vulgaris rubra* has emerged as a potential means to prevent and manage diseases associated with diminished NO bioavailability, notably arterial hypertension and endothelial dysfunction [24,25]. Beetroot contains phytochemical compounds that include ascorbic acid, carotenoids, phenolic acids, flavonoids, and highly bioactive pigments known as betalains (beetroot powder) characterized by their high antioxidant and anti-inflammatory activity [24,25]. Betalains have no ADI or MDI, but nitrates have been recommended for an ADI of 3.7 mg/kg [79]. Table 4 shows the RCTs conducted with beetroot.

Betalains also offer health-promoting properties, particularly for disorders characterized by chronic inflammation, such as CKD [24]. In this regard, a randomized crossover study [80] in CKD patients analyzed NO concentrations and renal resistance indexes as prognostic markers of CV mortality. Beetroot juice with a nitrate load of 300 mg was administered to the intervention group compared to the placebo. Hemodynamic parameters as well as plasma nitrate concentration and renal resistance indexes were measured before and 4h after treatment. The dietary nitrate exerted hypotensive effects on patients with diabetic nephropathy or hypertensive kidney disease without any changes in serum potassium and creatinine after they drank the beetroot juice. Likewise, the nitrate dietary load was able to decrease the renal resistance index—a marker of CKD progression and CV mortality. However, there are limited observations on beetroot, and further investigations are warranted. Therapeutic interventions directed toward the improvement of NO production, in addition to management of other CV risk factors, may prevent the development of endothelial dysfunction, in addition to facilitating the proper management of CKD patients at increased risk of CVD.

In the same way, a randomized single-blind placebo-controlled study carried out on HD patients [52] examined the kinetics of plasma nitrate. Eight HD patients and seven healthy volunteers were randomly assigned to receive 300 mL of nitrate-rich concentrated beetroot juice and a placebo. The beetroot juice consumption was safe and well-tolerated, with significantly higher plasma nitrate concentrations in HD patients. Long-term efficacy studies of dietary nitrate supplementation are warranted to investigate clinical benefits in subjects with reduced NO bioactivity, including CKD and HD patients.

Beetroot and its bioactive compounds (betalains, mostly), can modulate mechanisms related to CKD progression and CVD, indicating that it could be a promising nutritional therapy to modulate common CKD-associated complications, such as arterial hypertension, endothelial dysfunction, inflammation, and oxidative stress [81]. As a non-pharmacological intervention, beetroot juice may offer health benefits for CKD and dialysis patients, but further clinical trials are needed.

### 3.2. Potential Effects of Some Medicinal Plants on Chronic Kidney Disease

#### 3.2.1. Catechins from Green Tea (Camellia Sinensis)

Green tea comes from the plant Camellia sinensis and has high anti-inflammatory, antioxidant, and antimutagenic properties because of its polyphenolic compounds in various biological systems [26]. Furthermore, green tea is distinguished by the presence of catechins, a subgroup of polyphenols, known for their powerful antioxidant activities. Three main catechins have been documented in green tea, epicatechin, epigallocatechin, and epicatechin-3-gallate (EGCG). The catechin EGCG is the most abundant, with an ADI of <800 mg/day and an MDI of 90–300 mg/day obtained from green tea infusions [82]. 

The health effects of EGCG are mediated by the underlying molecular mechanisms, mainly by direct inhibition of stress- or stimulus-induced ROS overproduction. Moreover, ECGC can influence the Nrf2 complex, leading to nuclear translocation of free Nrf2, which subsequently binds to the antioxidant response element within the promoter region of cytoprotective genes encoding antioxidant enzymes, which are also modulated by the NF-κβ and TNF-α signaling pathways [27]. Table 5 shows the RCTs conducted with green tea or EGCG.

Various studies [53,54,55,83] have investigated the relationship between green tea and the mechanisms of CKD. An epidemiological study with Mendelian randomization in CKD researched the potential causal effects of tea intake on albuminuria and GFR [83]. The genome-wide association study summary data set for tea consumption was based on the UK Biobank, and the data on habitual tea consumption were obtained as a baseline from a dietary questionnaire. A total of 2672 single-nucleotide polymorphisms associated with tea consumption were found, 45 of which were independent and usable in CKD gen. Drinking an additional daily cup of tea had a nephroprotective effect by increasing GFR. Alternatively, high tea consumption was also inversely correlated with a lower risk of albuminuria. The study provided new hypotheses about green tea and CKD that should be explored in future clinical trials.

HD patients suffer from high levels of oxidative stress and antioxidant-decreased enzyme activity, e.g., SOD, CAT, and GSH-Px [84]. A clinical trial [53] studied the combination of EGC and Gand Amla extract (AE) in thirteen diabetic HD patients compared to healthy volunteers. The EGCG/AE enhanced antioxidant defense in diabetic HD patients. In addition, no hepatic or renal toxicity, and no inflammatory response were observed. The study showed that catechins improve antioxidant defenses, plasma glucose, CRP, and atherogenic indices (HDL and LDL/HDL ratio) in diabetic HD patients.

Likewise, another clinical study [54] studied the effect of supplementation with 800 mg of EGCG in treating residual albuminuria in 42 patients with diabetic nephropathy. The consumption of green tea significantly reduced albuminuria by diminishing podocyte apoptosis through activation of the wingless pathway. Also, a randomized placebo-controlled trial [55] evaluated the effects of supplementation with decaffeinated green tea extract on induced ROS and proinflammatory cytokines as CV risk factors in HD patients. The study consisted of three phases. Firstly, the researchers measured the plasma catechin concentrations in 6 healthy subjects and 10 HD patients after a single oral dose (455 mg of decaffeinated green tea extracts) that was comparable to four cups of green tea. In the following phase, the antioxidant capacity of catechins (455 mg) was compared with oral vitamin C (500 mg) in 10 HD patients. Lastly, the sample was randomized in a 7-month interventional study, in which 30 patients received a placebo and 14 patients received catechins (455 mg/d) from the third to the fifth month. Supplementation with catechins was more effective than vitamin C by reducing HD-enhanced plasma hypochlorous acid count activities and restoring paraoxonase-1 activity. Furthermore, the post-dialysis plasma concentrations of proapoptotic mediators, proinflammatory cytokines, and leukocyte-mediated anti-inflammatory molecules (the Fas/TNF receptor superfamily, IL-6R, IL-8, NAP-2, IL-1ra, IL-6R, IL-6R, IL-8, NAP-2, and IL-1ra) were downregulated significantly by the catechins. During the three experimental months, the pre-dialysis plasma concentrations of phosphatidylcholine hydroperoxide, TNF-α, sICAM-1, MCP-1, and CRP in the intervention group were lower than their baseline values and those in the control group. This study provided support for the possibility that catechins reduce HD-induced production of hydrogen peroxide and hypochlorous acid, CV risk factors, and proinflammation.

Previous preclinical evidence has suggested that EGCG may exert its protective effects through various mechanisms, including activation of Nrf2/HO-1 signaling, anti-inflammatory activity, and prevention of epithelial-to-mesenchymal transition [85]. Understanding the molecular interactions of EGCG with proteins, as well as its impact on biological processes at the cellular level, could provide valuable insights for the development of potential therapeutic interventions for CKD and dialysis patients. Moreover, causal research on the association between tea consumption and kidney function, particularly CKD, remains limited. This highlights the need for further investigation into the causal relationship between tea consumption, catechin intake, and kidney function, providing a compelling direction for future research in this area.

#### 3.2.2. Rhein, Emodin, and Aloe-Emodin from Rhubarb

In Asia, especially in Japan, not only traditional herbologists but also nephrologists have occasionally used herbal medicines for the treatment of CKD patients as part of a multi-approach therapy. *Rheum officinale* has long been used by practitioners of traditional Chinese medicine for its strong cathartic action and, more recently, to delay the progression of CKD. Identified active constituents of processed *Rheum officinale* include rhein, emodin, and aloe-emodin, each of which confers different pharmacologic actions [9]. In CKD, rhubarb contributes to the elimination of nitrogenous waste products through the gastrointestinal tract and the regulation of water and electrolyte balance. It has been reported that the abnormal expression of aquaporins could lead to less absorption of water in the colon or more secretion of intestinal juice. Emodin inhibits the genetic transcription and translation of the aquaporin 2 gene and decreases the gluconeogenesis of renal tubular cells and the adenosine triphosphate (ATP) content of epithelial mitochondria [56].

To date, a few studies that used rhubarb in CKD patients have been reported (Table 5). A randomized clinical trial [56] was conducted on 144 patients with CKD stage 3–4 to evaluate the efficacy and safety of rhubarb supplementation. Patients received conservative CKD management along with a rhubarb capsule (350 mg/thrice daily) or a placebo for 12 weeks. The results showed a reduction in levels of glucose, urea, creatinine, and 24 h total urine protein, whereas hemoglobin and GFR were increased. As for side effects, there was no statistical difference between the two groups.

The findings on rhubarb or *Rheum officinale* seem promising, but the lack of studies means that it not possible yet to draw strong conclusions about its use in patients with CKD.

#### 3.2.3. Astragalus Membranaceus (Huangqi)

Astragalus, known as Huangqi in China, a perennial herb in the Fabaceae family, is widely used in traditional Chinese medicine. The dried root of this plant is traditionally used as a tonic herb in CKD with a GFR less than 60 mL/min/1.73 m^2^. More than 100 compounds have been isolated and identified from Astragalus, such as flavonoids, saponins, polysaccharides, amino acids, and other compounds (ribobioside, sphingolipids, oligosaccharides, acids, enzymes, and trace elements).

Huangqi has been demonstrated to exhibit immunomodulatory effects, anti-inflammatory properties, and antioxidant activities [28,29] (Table 1 and Table 5). Various studies [86,87] have shown that Huangqi extracts modulate either pro- or anti-inflammatory immune responses depending on doses and cultural conditions. Administration of Huangqi extracts to palmitate-treated macrophages decreased the expression of proinflammatory cytokines and enhanced the production of anti-inflammatory cytokines. Similarly, Huangqi treatment was shown to promote T-helper (Th) cell differentiation toward Th1 and Th2 cell types [88]. In a recent metanalysis [89], the use of *Astragalus membranaceus* was associated with decreased plasma IL-6.

A controlled before-and-after clinical trial [57] studied the effect of *Astragalus membranaceus* on GFR in patients with advanced CKD stage 4–5 to assess the efficacy of *Astragalus membranaceus* in CKD progression combined with conventional therapy. Thirty-five CKD patients were treated with 2.5 g Astragalus twice a day, together with conventional therapy for more than 3 months. The supplementation of astragalus significantly increased the GFR in patients with CKD stage 4 during the first three months and maintained the GFR during the remaining eight months. The supplementation with Astragalus was effective in delaying CKD progression in this study, but new RCTs are needed to confirm the effects of this herbal therapy.

The effects of Astragalus have also been studied in PD patients. A retrospective single-center case–control study [88] measured the effects of astragalus on residual renal function and total daily urine volume. Eight PD patients and sixteen controls were included in this analysis. The *Astragalus membranaceus* formulae were ingested as decoctions prepared from raw herbs or concentrated Chinese medicine granules. Results showed that the supplemented group had significantly higher daily urine volumes and residual renal function at 2 years. The findings of the study highlighted the potential of *Astragalus membranaceus* in maintaining residual renal function in PD patients, but causal relationships cannot be established given the methodological design of the study.

Currently, there are significant adverse consequences associated with the utilization of Astragalus, including allergic response and gastrointestinal disorders [90]. However, further well-designed studies, particularly RCTs, are warranted to confirm these findings and elucidate the underlying mechanisms. 

#### 3.2.4. Triptolide from *Tripterygium Wilfordii* Hook F 

*Tripterygium wilfordii* Hook F (TwHF) is a perennial herb belonging to the family Celastraceae, and it is a traditional Chinese herbal medicine commonly used to treat inflammatory diseases [30]. The active products of TwHF include a variety of alkaloids, flavonoids, sterols, phenols, glycosides, triterpenoids, and proteins. Triptolide is one of the bioactive compounds in TwHF that has been implicated in the suppression of T cell proliferation and the induction of T cell apoptosis in the development of type 1 DM [31]. 

Nevertheless, research into the relationship between TwHF and CKD has been scarce (Table 1 and Table 5). A randomized clinical trial [58] was conducted to evaluate the efficacy and safety of TwHF for reducing proteinuria in sixty-five patients with diabetic nephropathy. Patients were given 120 mg of TwHF per day for three months, followed by 60 mg of TwHF/day for the remaining 3 months or 160 mg of valsartan daily for 6 months. Proteinuria in the TwHF group decreased at 1, 3, and 6 months. In contrast, in the valsartan group, proteinuria was not significantly attenuated at 1, 3, or 6 months post-intervention.

A systematic review of TwHF-derived treatments for CKD and dialysis patients is warranted. Various bioactive compounds in TwHF can act on CKD using a multitargeting pattern, thus providing insight into understanding herbal medicines and their mechanisms of action. However, the lack of clinical trials and the consistency of the results highlight that more studies are still needed to ensure the efficacy and safety of TwHF extract as an herbal medicine in CKD.

## 4. Conclusions

Considering that the rising prevalence of CKD imposes a significant burden on global healthcare systems due to high medical costs, incorporating nutrition-based therapies, such as those involving plant bioactive compounds, could serve as an affordable and manageable supplementary treatment for CKD and dialysis patients. Turmeric acts as an antioxidant and anti-inflammatory agent, and a potential modulator of the microbiota in CKD stages 3 and 4 and in HD patients. Likewise, propolis was shown to improve proteinuria in stage 2–4 CKD, as well as anti-inflammatory and antioxidant effects in dialysis patients. Additionally, green tea, as a medicinal plant, had antioxidant and anti-inflammatory effects in CKD and dialysis patients. This systematic review highlights the current impact of certain bioactive compounds and medicinal plants on the management and progression of CKD and in dialysis patients.

Many of these bioactive compounds have been found to mitigate inflammation by modulating the NF-κβ pathway, which lowers levels of cytokines and pro-inflammatory compounds. Others exhibit direct ROS scavenging properties due to their molecular structure or have indirect antioxidative effects through the upregulation of antioxidant enzymes. A diet tailored to CKD that includes natural foods containing these compounds may delay disease progression and quality of life while reducing health damage and economic costs related to complications. 

Based on these studies, traditional Chinese medicine could play a role in a comprehensive approach to managing CKD and dialysis patients. However, there remains insufficient evidence to endorse its use in clinical practice. Further clinical and observational studies with larger patient populations are therefore needed to better understand the effects of bioactive compounds and medicinal plants, the safe and effective dosages, as well as possible side effects in larger trials.

## Figures and Tables

**Table 1:** Bioactive compounds and food sources of plant-based foods and medicinal plants.

Bioactive Compounds from Plant-Based Foods	Alimentary Source	Therapeutic Effects	Refs.
Curcumin	Turmeric (spice)	Anti-inflammatory and antioxidant agent Microbiota modulator	[11,12,13,14]
Propolis (flavonoids, terpenes)	Resin obtained by bees	Anti-inflammatory, anti-proteinuric, and renal-protective activities	[15,16,17]
Sulforaphane	Broccoli, brussels sprouts, cauliflower, cabbage	Activates Nrf2 gene expression, leading to an antioxidant and anti-inflammatory effect	[18,19,20]
Genistein	Soy, red clover	Anti-inflammatory and improves nutritional status	[21,22]
Allicin	Garlic	Hypoglycemic, lipid-lowering agent, antioxidant and anti-inflammatory	[23]
Betalain	Beetroot	Reduction in blood pressure and anti-inflammatory	[24,25]
**Bioactive compounds from Medicinal plants**	**Medicinal Plants**		
Catechins (polyphenols)	Green tea (*Camelia sinensis*)	Anti-inflammatory and antioxidant effects	[26,27]
Rhein, emodin, and aloe-emodin	Rhubarb	Anti-inflammatory, antioxidant, antibacterial, and lipid-lowering agent	[9]
Flavonoids, polysaccharides, saponins, amino acids, and other compounds (riboside, sphingolipids, oligosaccharides, acids, enzymes, and trace elements)	*Astragalus membranaceus*	Hepatoprotective, diuretic, and expectorant properties Anti-inflammatory, antioxidant, and antiviral activities Renoprotective	[28,29]
Triptolide	*Tripterygium wilfordii* Hook F	Immunosuppressive and anti-inflammatory Renoprotective	[30,31]

**Table 2:** Potential effects of curcumin on chronic kidney disease and dialysis patients.

Bioactive Compound	Study Design and Population	Intervention	Molecular Mechanisms	Therapeutic Effect	Major Findings	Refs.
Curcumin	Randomized double-blind placebo-controlled clinical trial *n* = 101 CKD patients	Curcumin (107 mg) in each meal (320 mg/day) or placebo (starch), for 8 weeks	Cellular effect directly against ROS or indirectly via the Nrf2 pathway	Antioxidant agent	The curcumin-supplemented group showed improved ROS scavenging activity and MDA and decreased oxidative stress	[14]
	Randomized double-blind placebo-controlled clinical trial *n* = 28 HD patients	Juice containing 100 mL of orange juice, 12 g of carrot, and 2.5 g of turmeric (95% curcumin), 3 times a week for 12 weeks	Modulates the expression of mRNA to NF-kβ, Nrf2, and IL-1β	Anti-inflammatory agent Promising gut microbiota modulator	Significant reduction in NF-kβ mRNA expression and CRP levels in the curcumin group Significant decrease in gut-uremic toxins (PCS) Modulation of the intestinal microbiota	[13,39]
	Randomized double-blind clinical trial *n* = 119 HD patients	Turmeric (1 capsule with each meal containing 500 mg turmeric, of which 22.1 mg was the active ingredient curcumin; 3 capsules daily) Controls received starch capsules for the same 8 weeks	Inhibition of the NF-kβ cell signaling pathway; decreases MCP-1 by inhibiting IL-8 and IL-12 Increases the levels of antioxidant enzymes (GPx, GR, and CAT)	Anti-inflammatory agent Antioxidant agent	Significant reduction in plasma IL-6 and TNF-α concentrations compared with the placebo group CAT enzyme levels were increased in both groups significantly Significant elevation of albumin levels in the turmeric group	[43,44]
	Randomized double-blind placebo-controlled clinical trial *n* = 43 HD patients	Curcuminoid capsules (1 g/day; each capsule contained 167 mg of curcuminoids and microcrystalline cellulose as an excipient) or placebo (only corn starch) for 12 weeks	Antioxidant by activating Nrf2 pathway Anti-inflammatory via NF-kβ modulation	Antioxidant, eliminating ROS Anti-inflammatory agent	Enhancement or preservation of enzymatic antioxidant capacity (CAT, GPx, and GR) No changes in MDA and CRP concentrations	[12]
	Randomized double-blind placebo-controlled clinical trial *n* = 60 CKD stage 3–4 patients	Curcumin or placebo (starch) capsules of 500 mg, 3 times/day, from two days before the procedure to three days after	Anti-inflammatory via NF-kβ modulation Antioxidant by activating Nrf2 pathway	Anti-inflammatory agent Antioxidant effects	No changes were observed with curcumin supplementation, with no adverse effects of CIN	[40]
	Randomized double-blind placebo-controlled clinical trial *n* = 24 CKD stage 3a–4 patients	Meriva^®^ 500 mg tablet, twice a day (curcumin (500 mg) film-coated tablets containing 100 mg of highly bioavailable curcuminoids) for 3 or 6 months	Reduction pro-inflammatory mediators (MCP-1, IFN-γ, and IL-4) and lipid peroxidation Increasing beneficial microbial diversity and reduction in uremic toxins (IS and PCS) in the gut barrier	Anti-inflammatory agent Microbiota modulator	Significant reduction in pro-inflammatory mediators MCP-1, IFN-γ, and IL-4 Lipid peroxidation markers, such as TBARS, were associated with curcumin intervention Improvement in gut diversity at the phylum level (Increase *Lachnoclostridium* spp.; Decrease *Escherichia* spp. and *Shigella* spp.)	[45]

CAT, Catalase enzyme; CIN, Contrast-induced nephropathy; CRP, C-reactive protein; GPx, Glutathione peroxidase; IFN-γ, Interferon-γ; IL-n, Interleukin; IS, Indoxyl-sulfate; MDA, Malonaldehyde; NF-kβ, Nuclear factor kappa-B; Nrf2, Nuclear factor erythroid 2-related factor 2; PCS, p-cresyl sulfate; ROS, Reactive oxygen species; TBARS, Thiobarbituric acid reactive substances; TNF-α, Tumor necrosis factor-α.

**Table 3:** Potential effects of propolis and sulforaphane in chronic kidney disease and dialysis patients.

Bioactive Compound	Study Design and Population	Intervention	Molecular Mechanisms	Therapeutic Effect	Major Findings	Refs.
Propolis	Randomized double-blind placebo-controlled study *n* = 32 CKD stage 2–4 patients	500 mg/day (4 tablets of 125 mg each twice a day) or placebo (starch) for 12 months	Decreasecytokine MCP-1 promotes the recruitment of monocytes and their transformation into macrophages	Proteinuria reduction Improves renal function	Propolis reduced proteinuria Significant reduction urinary excretion of MCP-1	[41]
	Randomized double-blind placebo-controlled study *n* = 41 HD patients	Propolis: 4 capsules of 100 mg/day containing EPP-AF^®^ green propolis extract) or placebo (4 capsules of 100 mg/day from starch) for 2 months	Anti-inflammatory via NF-kβ modulation	Anti-inflammatory agent	↓ TNF-α and MIP-1β	[46]
	Randomized double-blind placebo-controlled trial *n* = 19 PD patients	4 capsules of 500 mg/day containing EPP-AF^®^ green propolis extract or placebo from starch (4 capsules of 500 mg/day) for 2 months	Anti-inflammatory activation via Nrf2 and inhibition via NF-kβ	Anti-inflammatory agent	DecreaseTNF-α after propolis intervention No significant changes in MDA, IL-6, or CRP	[47]
Sulforaphane	Randomized double-blind crossover study *n* = 30 HD patients	150 μmol of sulforaphane (extract 1.0% powder with 0.5% myrosinase) or placebo (starch) for 2 months	An agonistic action of Nrf2 influences the transcription of antioxidant enzymes and inhibits NF-kβ activity, leading to negative regulation of proinflammatory genes and the inflammasome	Antioxidant agent Anti-inflammatory agent	No changes in antioxidants (Nrf2 and MDA) and anti-inflammatory effects (NF-kβ mRNA expression)	[42]
	Randomized placebo-controlled clinical trial *n* = 25 CKD stages 3–5 patients	400 μg of L-sulforaphane daily or placebo (400 μg of cornstarch) for 1 month	Nrf2 pathway stimulates the expression of a series of antioxidants and anti-inflammatory genes, including NQO1, GPx, and SOD	Antioxidant agent Cardiovascular benefits	Significant increase in the mRNA expression of Nrf2 and NQO1 Decrease hosphate, glucose, and triglyceride levels	[48]

CRP, C-reactive protein; GPx, Glutathione peroxidase; MCP-1, Monocyte chemotactic protein 1; MDA, Malonaldehyde; MIP-1β, Macrophage inflammatory protein-1β; NF-kβ, Nuclear factor *kappa*-β; Nrf2, Nuclear factor erythroid 2-related factor 2; NQO1, NADPH: quinone oxidoreductase; SOD, Superoxide dismutase; TNF-α, Tumor necrosis factor-α.

**Table 4:** Potential effects of genistein, allicin, and beetroot in chronic kidney disease and dialysis patients.

Bioactive Compound	Study Design and Population	Intervention	Molecular Mechanisms	Therapeutic Effect	Major Findings	Refs.
Genistein	Randomized double-blind controlled pilot study *n* = 32 HD patients with underlying systemic inflammation	Soy protein isolate (protein drink, 54 mg isoflavones), during each HD session, and a protein snack bar or a cereal-like breakfast product (26 mg isoflavones) on each non-dialysis day for 8 weeks	Increasing IGF-1, a potent anabolic hormone which results in Reduction of protein degradation and Increase protein synthesis	Anti-inflammatory agent Improved nutritional status	Isoflavones reduced inflammatory biomarkers (CRP) and improved nutritional indicators (IGF-1, albumin)	[49]
Allicin	Randomized double-blind clinical trial *n* = 70 HD patients	Group intervention: 300 mg of garlic powder (containing 1.3 mg of the garlic extract) vs. placebo for 8 weeks After a 6-week wash-out period, the agents were switched between groups	Decrease cytokine production in endothelial cells, modifying adipocyte metabolic profile and stimulating anti-inflammatory gene expression	Cardioprotective Antioxidant agent	Garlic extract was useful in reducing triacylglyceride, oxidized LDL, and homocysteine levels	[50]
	Randomized double-blind parallel-designed clinical trial *n* = 40 PD patients	400 mg of standardized garlic extract (tablets containing 1000 µg of Alliin) twice a day for 8 weeks	Decrease cytokine production in endothelial cells, creating an anti-inflammatory gene expression profile and modifying adipocyte metabolic profile	Anti-inflammatory agent Lipid-lowering agent	Significant decrease in IL-6, CRP, and erythrocyte sedimentation rates in the intervention group Significant decreasein IL-6 with placebo	[51]
Beetroot	Randomized single-blind placebo-controlled study *n* = 8 HD patients	300 mL of nitrate-rich concentrated beetroot juice or placebo (<0.01 mmol/L nitrate; 70 mL), in a crossover manner, with a washout period of at least seven days between each trial	Modulation of the activity of the nitrate–nitrite–NO pathway	Vasodilator effects	Significantly ↑ plasma nitrate modulates arterial blood flow and blood pressure	[52]

CRP, C-reactive protein; IGF-1, Insulin-like growth factor; IL-6, Interleukin-6; LDL, Low-density lipoprotein; NO, Nitric oxide.

**Table 5:** Potential effects of bioactive compounds from medicinal plants in chronic kidney disease and dialysis patients.

Bioactive Compound	Study Design and Population	Intervention	Molecular Mechanisms	Therapeutic Effect	Major Findings	Refs.
Green tea (Camellia sinensis)	Randomized double-blind controlled study *n* = 13 diabetic HD patients	100 mg of EGCG and100 mg of AE for 3 months	Antioxidant effects by activating the Nrf2 pathway and ROS scavengers	Antioxidant Anti-inflammatory Cardioprotective	Improves antioxidant defenses (plasma FRAP, and increaseplasma polyphenols) Decrease CRP and improve atherogenic index (LDL/HDL ratio)	[53]
	Randomized double-blind placebo-controlled study n = 42 diabetic nephropathy patients	800 mg of EGCG or placebo for 12 weeks	Oxidative stress modulated by inhibiting the NOX4 protein pathway and NOS enzymes Uncoupling and reducing podocyte apoptosis by activating the Wnt pathway	Antioxidant Anti-inflammatory Decreases podocyte apoptosis	Decrease DKK-1, a WNT pathway inhibitor, post-intervention Decrease TNF-α and CRP Reduction Albuminuria	[54]
	Randomized double-blind study *n* = 44 HD patients	455 mg of decaffeinated green tea extracts (4 cups of green tea) or placebo for 7 months	Modulation of ROS (hydrogen peroxide and hypochlorous acid) by oxidizing LDLs Inhibits proinflammatory and proapoptotic oxidative injury via decreasing of the production of ROS, translocation of NF-kβ and activated protein, and the endothelial adhesion molecule ICAM-1	Antioxidant Cardioprotective Anti-inflammatory	Decreaseof HD-induced plasma hydrogen peroxide activity and lower hypochlorous acid activity (ROS) Decrease phosphatidylcholine, hydroperoxide), sICAM-1, and MCP-1 Decreaseserum CRP, TNF-α-, and IL-1ra by inhibitory effect on HD-enhanced leukocyte activation	[55]
Rhubarb (Rheum oficinal)	Randomized clinical trial *n* = 144 CKD stage 3–4 patients	Group intervention: conservative CKD treatment + rhubarb capsule (350 mg) thrice daily Controls: conservative CKD treatment + telmisartan (40 mg/day) Duration: 12 weeks	Emodin inhibits the transcription and translation of the AQP2 gene Rhein inhibits TGF-*β*1 and fibronectin expression in renal tissue	Cardioprotective Antiproteinuric Renoprotective	Systolic and diastolic blood pressure reduction in rhubarb intervention Decrease24 h total urine protein and improved GFR after 12 weeks post-intervention Decreasepotassium in both groups	[56]
*Astragalus* *membranaceus* (Huangqi)	Prospective study, clinical trial *n* = 35 CKD stage 4–5 patients	2.5 g *Astragalus* *membranaceus* twice a day, together with conventional therapy for 3 months	Enhanced NO production via activation of endothelial NO synthase and scavenging ROS Improves renal function by reducing NF-kβmRNA levels in the renal cortex	Antiproteinuric Renoprotective	Reduction 24 h total urine protein In CKD stage 4, the GFR increased at 3 months and remained stable during the following 12 months In CKD stage 5, GFR increased slightly at 3 months, declining at 6 and 12 months	[57]
*Tripterygium* *wilfordii* Hook F	Randomized controlled trial *n* = 65 diabetic nephropathy patients	TwHF: 120 mg/day, followed by 60 mg/day of TwHF for 3 months Valsartan group: 160 mg of valsartan daily for 6 months	Inhibits the expression of certain inflammatory cytokines (TNF-α, IL-1β, IL-6, and IFN-γ)	Antiproteinuric Renoprotective	TwHF group: Reduction proteinuria at 1, 3, and 6 months Valsartan group: proteinuria was not reduced at 1, 3, or 6 months post-intervention DecreaseGFR in the valsartan group vs. the TwHF group (26.4% vs. 13.7%, respectively)	[58]

AE, Gand Amla extract; CRP, C-reactive protein; DKK-1, Dickkopf WNT pathway inhibitor 1; EGCG, Epigalocatequin; FRAP, Ferric reducing antioxidant power; GFR, Glomerular filtration rate; IFN-γ, Interferon-γ; ICAM-1, intercellular adhesion molecule -1; IL-n, Interleukin; IL-1ra, Interleukin-1ra; IL-1β, Interleukin-1β; LDL, Low-density lipoprotein; MCP-1, Monocyte chemotactic protein 1; NF-kβ, Nuclear factor *kappa*-β; NOS, Nitric oxide synthetase; NOX4, NADPH oxidase 4 pathway; Nrf2, Nuclear factor erythroid 2-related factor 2; NO, Nitric oxide; ROS, Reactive oxygen species; TGF-*β*1, transforming growth factor-beta 1;TNF-α, Tumor necrosis factor-α; Wnt, Wingless-related integration site.

## Data Availability

Not applicable.

## References

[1] Hill N.R., Fatoba S.T., Oke J.L., Hirst J.A., O’Callaghan C.A., Lasserson D.S., Hobbs F.D. (2016). Global prevalence of chronic kidney disease—A systematic review and meta-analysis. PLoS ONE.

[2] Foreman K.J., Marquez N., Dolgert A., Fukutaki K., Fullman N., McGaughey M., Pletcher M.A., Smith A.E., Tang K., Yuan C.W. (2018). Forecasting life expectancy, years of life lost, and all-cause and cause-specific mortality for 250 causes of death: Reference and alternative scenarios for 2016–2040 for 195 countries and territories. Lancet.

[3] Kovesdy C.P. (2022). Epidemiology of chronic kidney disease: An update. Kidney Int..

[4] Kris-Etherton P.M., Lefevre M., Beecher G.R., Gross M.D., Keen C.L., Etherton T.D. (2004). Bioactive compounds in nutrition and health-research methodologies for establishing biological function: The antioxidant and anti-inflammatory effects of flavonoids on atherosclerosis. Annu. Rev. Nutr..

[5] Quintela B.C.S.F., Carioca A.F., de Oliveira J.G.R., Fraser S.D., da Silva Junior G.B. (2021). Dietary patterns and chronic kidney disease outcomes: A systematic review. Nephrology.

[6] Biesalski H.K., Dragsted L.O., Elmadfa I., Grossklaus R., Müller M., Schrenk D., Walter P., Weber P. (2009). Bioactive compounds: Definition and assessment of activity. Nutrition.

[7] Owen L., Corfe B. (2017). The role of diet and nutrition on mental health and wellbeing. Proc. Nutr. Soc..

[8] Gantenbein K.V., Kanaka-Gantenbein C. (2021). Mediterranean diet as an antioxidant: The impact on metabolic health and overall wellbeing. Nutrients.

[9] Wang H., Song H., Yue J., Li J., Hou Y.B., Deng J.L. (2012). Rheum officinale (a traditional Chinese medicine) for chronic kidney disease. Cochrane Database Syst. Rev..

[10] Gámez-Villazana J. (2020). Advances in the determination of bioactive compounds in foods. Cienc. Tecnol. Agrollanía.

[11] Martins-de-Souza D. (2021). Studies on Biomarkers and New Targets in Aging Research in Iran Focus on Turmeric and Curcumin.

[12] Rodrigues H.C., Martins T.F.P., Santana N.C.e.S., Braga C.C., Silva M.C., da Cunha L.C., Sugizaki C.S.A., Freitas A.T.V.S., Costa N.A., Peixoto M.D.R.G. (2021). Antioxidant and anti-inflammatory response to curcumin supplementation in hemodialysis patients: A randomized, double-blind, placebo-controlled clinical trial. Clin. Nutr. ESPEN.

[13] Salarolli R.T., Alvarenga L., Cardozo L.F.M.F., Teixeira K.T.R., de Moreira L.S.G., Lima J.D., Rodrigues S.D., Nakao L.S., Fouque D., Mafra D. (2021). Can curcumin supplementation reduce plasma levels of gut-derived uremic toxins in hemodialysis patients? A pilot randomized, double-blind, controlled study. Int. Urol. Nephrol.

[14] Jiménez-Osorio A.S., García-Niño W.R., González-Reyes S., Álvarez-Mejía A.E., Guerra-León S., Salazar-Segovia J., Falcón I., Montes de Oca-Solano H., Madero M., Pedraza-Chaverri J. (2016). The effect of dietary supplementation with curcumin on redox status and Nrf2 activation in patients with nondiabetic or diabetic proteinuric chronic kidney disease: A pilot study. J. Ren. Nutr..

[15] Gheflati A., Dehnavi Z., Yazdi A.G., Khorasanchi Z., Raeisi-Dehkordi H., Ranjbar G. (2021). The effects of propolis supplementation on metabolic parameters: A systematic review and meta-analysis of randomized controlled clinical trials. Avicenna J. Phytomed..

[16] Forma E., Bryś M. (2021). Anticancer activity of propolis and its compounds. Nutrients.

[17] Zulhendri F., Lesmana R., Tandean S., Christoper A., Chandrasekaran K., Irsyam I., Suwantika A.A., Abdulah R., Wathoni N. (2022). Recent Update on the Anti-Inflammatory Activities of Propolis. Molecules.

[18] Cardozo L.F.M.F., Alvarenga L.A., Ribeiro M., Dai L., Shiels P.G., Stenvinkel P., Lindholm B., Mafra D. (2021). Cruciferous vegetables: Rationale for exploring potential salutary effects of sulforaphane-rich foods in patients with chronic kidney disease. Nutr. Rev..

[19] Vanduchova A., Anzenbacher P., Anzenbacherova E. (2019). Isothiocyanate from Broccoli, Sulforaphane, and Its Properties. J. Med. Food.

[20] Houghton C.A. (2019). Sulforaphane: Its “Coming of Age” as a Clinically Relevant Nutraceutical in the Prevention and Treatment of Chronic Disease. Oxid. Med. Cell. Longev..

[21] Helen K., Peterson T.G., Barnes S. (1998). Mechanisms of action of the soy isoflavone genistein: Emerging role for its effects via transforming growth factor beta signaling pathways. Am. J. Clin. Nutr..

[22] Peng Q., Li Y., Shang J., Huang H., Zhang Y., Ding Y., Liang Y., Xie Z., Chen C. (2022). Effects of Genistein on Common Kidney Diseases. Nutrients.

[23] Soroka N., Silverberg D.S., Greemland M., Birk Y., Blum M., Peer G., Iaina A. (1998). Comparison of a Vegetable-Based (Soya) and an Animal-Based Low-Protein Diet in Predialysis Chronic Renal Failure Patients. Nephron.

[24] Clifford T., Howatson G., West D.J., Stevenson E.J. (2015). The potential benefits of red beetroot supplementation in health and disease. Nutrients.

[25] Griffiths A., Alhulaefi S., Hayes E.J., Matu J., Brandt K., Watson A. (2023). Exploring the Advantages and Disadvantages of a Whole Foods Approach for Elevating Dietary Nitrate Intake: Have Researchers Concentrated Too Much on Beetroot Juice?. Appl. Sci..

[26] Avila-Carrasco L., García-Mayorga E.A., Díaz-Avila D., Garza-Veloz I., Martinez-Fierro M.L., González-Mateo G.T. (2021). Potential therapeutic effects of natural plant compounds in kidney disease. Molecules.

[27] Kanwar J., Taskeen M., Mohammad I., Huo C., Chan T.H., Dou Q.P. (2012). Recent advances on tea polyphenols. Front. Biosci..

[28] Fu J., Wang Z., Huang L., Zheng S., Wang D., Chen S., Zhang H., Yang S. (2014). Review of the botanical characteristics, phytochemistry, and pharmacology of Astragalus membranaceus (Huangqi). Phytother. Res..

[29] Ji B., Xuan L., Zhang Y., Zhang G., Meng J., Mu W., Liu J., Paek K.Y., Park S.Y., Wang J. (2023). Advances in Biotechnological Production and Metabolic Regulation of Astragalus membranaceus. Plants.

[30] Song C.Y., Xu Y.G., Lu Y.Q. (2020). Use of Tripterygium wilfordii Hook F for immune-mediated inflammatory diseases: Progress and future prospects. J. Zhejiang Univ. Sci. B.

[31] Sravanthi V., Sampath Kumar H.M., Brahmachari G. (2017). Immunomodulators in Prophylaxis and Therapy of Type-1 Diabetes. Discovery and Development of Antidiabetic Agents from Natural Products.

[32] Khan M.A., Kassianos A.J., Hoy W.E., Alam A.K., Healy H.G., Gobe G.C. (2022). Promoting Plant-Based Therapies for Chronic Kidney Disease. J. Evid. Based. Integr. Med..

[33] Carrero J.J., González-Ortiz A., Avesani C.M., Bakker S.J.L., Bellizzi V., Chauveau P., Clase C.M., Cupisti A., Espinosa-Cuevas A., Molina P. (2020). Plant-based diets to manage the risks and complications of chronic kidney disease. Nat. Rev. Nephrol..

[34] Tabas I., Glass C.K. (2013). Anti-inflammatory therapy in chronic disease: Challenges and opportunities. Science.

[35] Rysz J., Franczyk B., Ławiński J., Olszewski R., Ciałkowska-Rysz A., Gluba-Brzózka A. (2021). The Impact of CKD on Uremic Toxins and Gut Microbiota. Toxins.

[36] Mafra D., Fouque D. (2015). Gut microbiota and inflammation in chronic kidney disease patients. Clin. Kidney J..

[37] Borges N.A., Barros A.F., Nakao L.S., Dolenga C.J., Fouque D., Mafra D. (2016). Protein-Bound Uremic Toxins from Gut Microbiota and Inflammatory Markers in Chronic Kidney Disease. J. Ren. Nutr..

[38] Jadad A.R., Moore R.A., Carroll D., Jenkinson C., Reynolds D.J.M., Gavaghan D.J., McQuay H.J. (1996). Assessing the quality of reports of randomized clinical trials: Is blinding necessary?. Control. Clin. Trials..

[39] Alvarenga L., Salarolli R., Cardozo L.F.M.F., Santos R.S., de Brito J.S., Kemp J.A., Reis D., de Paiva B.R., Stenvinkel P., Lindholm B. (2020). Impact of curcumin supplementation on expression of inflammatory transcription factors in hemodialysis patients: A pilot randomized, double-blind, controlled study. Clin Nut..

[40] Hami M., Bigdeli A., Khameneh-Bagheri R., Rajabi O., Salehi M., Zahedi-Avval F. (2019). The Effect of Curcumin in Prevention of Contrast Nephropathy Following Coronary Angiography or Angioplasty in CKD Patients. Iran. J. Kidney Dis..

[41] Silveira M.A.D., Teles F., Berretta A.A., Sanches T.R., Rodrigues C.E., Seguro A.C. (2019). Effects of Brazilian green propolis on proteinuria and renal function in patients with chronic kidney disease: A randomized, double-blind, placebo-controlled trial. BMC Nephrol..

[42] Ribeiro M., Cardozo L.F.M.F., Paiva B.R., Baptista B.G., Fanton S., Alvarenga L., Lima L.S., Britto I., Nakao L.S., Fouque D. (2023). Sulforaphane supplementation did not modulate NRF2 and NF-kB mRNA expressions in hemodialysis patients. J. Ren. Nut..

[43] Pakfetrat M., Akmali M., Malekmakan L., Dabaghimanesh M., Khorsand M. (2015). Role of turmeric in oxidative modulation in end-stage renal disease patients. Hemodial. Int..

[44] Samadian F., Dalili N., Gholi F.P.R., Fattah M., Malih N., Nafar M., Firoozan A., Ahmadpoor P., Samavat S., Ziaie S. (2017). Evaluation of Curcumin’s effect on inflammation in hemodialysis patients. Clin. Nutr. ESPEN.

[45] Pivari F., Mingione A., Piazzini G., Ceccarani C., Ottaviano E., Brasacchio C., Dei Cas M., Vischi M., Cozzolino M.G., Fogagnolo P. (2022). Curcumin Supplementation (Meriva®) Modulates Inflammation, Lipid Peroxidation and Gut Microbiota Composition in Chronic Kidney Disease. Nutrients.

[46] Chermut T.R., Fonseca L., Figueiredo N., de Oliveira Leal V., Borges N.A., Cardozo L.F., Correa Leite P.E., Alvarenga L., Regis B., Delgado A. (2023). Effects of propolis on inflammation markers in patients undergoing hemodialysis: A randomized, double-blind controlled clinical trial. Complement. Ther. Clin. Pract..

[47] Baptista B.G., Fanton S., Ribeiro M., Cardozo L.F., Regis B., Alvarenga L., Ribeiro-Alves M., Berretta A.A., Shiels P.G., Mafra D. (2023). The effect of Brazilian Green Propolis extract on inflammation in patients with chronic kidney disease on peritoneal dialysis: A randomised double-blind controlled clinical trial. Phytomedicine.

[48] Ribeiro M., Alvarenga L., Coutinho-Wolino K.S., Nakao L.S., Cardozo L.F., Mafra D. (2024). Sulforaphane upregulates the mRNA expression of NRF2 and NQO1 in non-dialysis patients with chronic kidney disease. Free Radic. Biol. Med..

[49] Fanti P., Asmis R., Stephenson T.J., Sawaya B.P., Franke A.A. (2006). Positive effect of dietary soy in ESRD patients with systemic inflammation—Correlation between blood levels of the soy isoflavones and the acute-phase reactants. Nephrol. Dial. Transplant..

[50] Asgharpour M., Khavandegar A., Balaei P., Enayati N., Mardi P., Alirezaei A., Bakhtiyari M. (2021). Efficacy of Oral Administration of Allium sativum Powder “garlic Extract” on Lipid Profile, Inflammation, and Cardiovascular Indices among Hemodialysis Patients. Evid. Based Complement. Alternat. Med..

[51] Zare E., Alirezaei A., Bakhtiyari M., Mansouri A. (2019). Evaluating the effect of garlic extract on serum inflammatory markers of peritoneal dialysis patients: A randomized double-blind clinical trial study. BMC Nephrol..

[52] Heredia-Martinez A., Rosa-Diez G., Ferraris J.R., Sohlenius-Sternbeck A.K., Nihlen C., Olsson A., Lundberg J.O., Weitzberg E., Carlström M., Krmar R.T. (2022). Plasma Nitrate and Nitrite Kinetics after Single Intake of Beetroot Juice in Adult Patients on Chronic Hemodialysis and in Healthy Volunteers: A Randomized, Single-Blind, Placebo-Controlled, Crossover Study. Nutrients.

[53] Chen T.S., Liou S.Y., Wu H.C., Tsai F.J., Tsai C.H., Huang C.Y. (2011). Efficacy of Epigallocatechin-3-Gallate and Amla (Emblica officinalis) Extract for the Treatment of Diabetic-Uremic Patients. J. Med. Food.

[54] Borges C.M., Papadimitriou A., Duarte D.A., Lopes De Faria J.M., Lopes De Faria J.B. (2016). The use of green tea polyphenols for treating residual albuminuria in diabetic nephropathy: A double-blind randomised clinical trial. Sci. Rep..

[55] Hsu S.P., Wu M.S., Yang C.C., Huang K.C., Liou S.Y., Hsu S.M., Chien C.T. (2007). Chronic green tea extract supplementation reduces hemodialysis-enhanced production of hydrogen peroxide and hypochlorous acid, atherosclerotic factors, and proinflammatory cytokines 1–3. Am. J. Clin. Nutr..

[56] Khan I.A., Nasiruddin M., Haque S.F., Khan R.A. (2014). Evaluation of Rhubarb Supplementation in Stages 3 and 4 of Chronic Kidney Disease: A Randomized Clinical Trial. Int. J. Chronic Dis..

[57] Okuda M., Horikoshi S., Matsumoto M., Tanimoto M., Yasui H., Tomino Y. (2012). Beneficial effect of Astragalus membranaceus on estimated glomerular filtration rate in patients with progressive chronic kidney disease. Hong Kong J. Nephrol..

[58] Ge Y., Xie H., Li S., Jin B., Hou J., Zhang H., Hi M., Liu Z. (2013). Treatment of diabetic nephropathy with Tripterygium wilfordii Hook F extract: A prospective, randomized, controlled clinical trial. J. Transl. Med..

[59] Zhang W., He J., Zhang F., Huang C., Wu Y., Han Y., Zhang W., Zhao Y. (2013). Prognostic role of C-reactive protein and Interleukin-6 in dialysis patients: A systematic review and meta-analysis. J. Nephrol..

[60] Cobo G., Lindholm B., Stenvinkel P. (2018). Chronic inflammation in end-stage renal disease and dialysis. Nephrol. Dial. Tranplant..

[61] Jankowska M., Cobo G., Lindholm B., Stenvinkel P. (2017). Inflammation and Protein-Energy Wasting in the Uremic Milieu. Contrib. Nephrol..

[62] Rysz J., Franczyk B., Ławiński J., Gluba-Brzózka A. (2020). Oxidative stress in ESRD patients on dialysis and the risk of cardiovascular diseases. Antioxidants.

[63] Rapa S.F., Di Iorio B.R., Campiglia P., Heidland A., Marzocco S. (2020). Inflammation and oxidative stress in chronic kidney disease—Potential therapeutic role of minerals, vitamins and plant-derived metabolites. Int. J. Mol. Sci..

[64] Vaziri N.D., Wong J., Pahl M., Piceno Y.M., Yuan J., Desantis T.Z., Ni Z., Nguyen T.H., Andersen G.L. (2013). Chronic kidney disease alters intestinal microbial flora. Kidney Int..

[65] Shi K., Wang F., Jiang H., Liu H., Wei M., Wang Z. (2014). Gut Bacterial Translocation May Aggravate Microinflammation in Hemodialysis Patients. Dig. Dis. Sci..

[66] Cabała S., Ożgo M., Herosimczyk A. (2024). The Kidney–Gut Axis as a Novel Target for Nutritional Intervention to Counteract Chronic Kidney Disease Progression. Metabolites.

[67] Shoba G., Joy D., Joseph T., Majeed M., Rajendran R., Srinivas P.S. (1998). Influence of Piperine on the Pharmacokinetics of Curcumin in Animals and Human Volunteers. Planta Med..

[68] Hegde M., Girisa S., BharathwajChetty B., Vishwa R., Kunnumakkara A.B. (2023). Curcumin Formulations for Better Bioavailability: What We Learned from Clinical Trials Thus Far?. ACS Omega.

[69] Alvarenga L., Cardozo L.F.M.F., Borges N.A., Chermut T.R., Ribeiro M., Leite M., Shiels P.G., Stenvinkel P., Mafra D. (2021). To bee or not to bee? The bee extract propolis as a bioactive compound in the burden of lifestyle diseases. Nutrition.

[70] Duarte Silveira M.A., Malta-Santos H., Rebouças-Silva J., Teles F., Batista Dos Santos Galvão E., Pinto De Souza S., Dantas Dutra F.R., Dantas Gomes M.M., Teixeira M.B., Miranda Rebelo da Conceição L.F. (2022). Effects of Standardized Brazilian Green Propolis Extract (EPP-AF®) on Inflammation in Haemodialysis Patients: A Clinical Trial. Int. J. Nephrol..

[71] Anvarifard P., Ostadrahimi A., Ardalan M., Anbari M., Ghoreishi Z. (2023). The effects of propolis on pro-oxidant–antioxidant balance, glycemic control, and quality of life in chronic kidney disease: A randomized, double-blind, placebo-controlled trial. Sci. Rep..

[72] Chavda V.P., Chaudhari A.Z., Teli D., Balar P., Vora L. (2023). Propolis and Their Active Constituents for Chronic Diseases. Biomedicines.

[73] Pasupuleti V.R., Sammugam L., Ramesh N., Gan S.H. (2017). Honey, Propolis, and Royal Jelly: A Comprehensive Review of Their Biological Actions and Health Benefits. Oxid. Med. Cell. Longev..

[74] Liebman S.E., Le T.H. (2021). Eat your broccoli: Oxidative stress, nrf2, and sulforaphane in chronic kidney disease. Nutrients.

[75] García E.L., Lesmes I.B., Perales A.D., Moreno V., Baquedano M.P.P., Velasco A.M.R., Salvo U.F., Romero L.T., Porcel F.B.O., Laín S.A. (2022). Informe del Comité Científico de la Agencia Española de Seguridad Alimentaria y Nutrición (AESAN) sobre recomendaciones dietéticas sostenibles y recomendaciones de actividad física para la población española. Rev. Com. Científico AESAN.

[76] Lin C.Y., Yao C.A. (2020). Potential role of NRF2 activators with dual antiviral and anti-inflammatory properties in the management of viral pneumonia. Infect. Drug. Resist..

[77] Ribeiro M., Alvarenga L., Cardozo L.F.M.F., Chermut T.R., Sequeira J., de Souza Gouveia Moreira L., Teixeira K.T.R., Shiels P.G., Stenvinkel P., Mafra D. (2021). From the distinctive smell to therapeutic effects: Garlic for cardiovascular, hepatic, gut, diabetes and chronic kidney disease. Clin. Nutr..

[78] Tuteja N., Chandra M., Tuteja R., Misra M.K. (2004). Nitric Oxide as a Unique Bioactive Signaling Messenger in Physiology and Pathophysiology. J. Biomed. Biotechnol..

[79] Reglamento (UE) 2023/915 de la Comisión (2023). Relativo a los Límites Máximos de Determinados Contaminantes en los Alimentos. https://www.boe.es/buscar/doc.php?id=DOUE-L-2023-80614.

[80] Kemmner S., Lorenz G., Wobst J., Kessler T., Wen M., Günthner R., Stock K., Heemann U., Burkhardt K., Baumann M. (2017). Dietary nitrate load lowers blood pressure and renal resistive index in patients with chronic kidney disease: A pilot study. Nitric Oxide.

[81] Moreira L.D.S.G., Fanton S., Cardozo L., Borges N.A., Combet E., Shiels P.G., Stenvinkel P., Mafra D. (2022). Pink pressure: Beetroot (Beta vulgaris rubra) as a possible novel medical therapy for chronic kidney disease. Nutr. Rev..

[82] (2022). Reglamento (UE) 2022/2340 de la Comisión Respecta a los Extractos de Té Verde que Contienen (-) 3-Galato de Epigalocatequina. https://www.boe.es/buscar/doc.php?id=DOUE-L-2022-81764.

[83] Zhang Y., Xiong Y., Shen S., Yang J., Wang W., Wu T., Chen L., Yu Q., Zuo H., Wang X. (2022). Causal Association Between Tea Consumption and Kidney Function: A Mendelian Randomization Study. Front. Nutr..

[84] Dursun E., Dursun B., Suleymanlar G., Ozben T. (2005). Effect of haemodialysis on the oxidative stress and antioxidants in diabetes mellitus. Acta Diabetol..

[85] Kanlaya R., Thongboonkerd V. (2019). Molecular Mechanisms of Epigallocatechin-3-Gallate for Prevention of Chronic Kidney Disease and Renal Fibrosis: Preclinical Evidence. Curr. Dev. Nutr..

[86] Zhong Y., Deng Y., Chen Y., Chuang P.Y., He J.C. (2013). Therapeutic use of traditional Chinese herbal medications for chronic kidney diseases. Kidney Int..

[87] Huang K., Zhang P., Zhang Z., Youn J.Y., Wang C., Zhang H. (2021). Traditional Chinese Medicine (TCM) in the treatment of COVID-19 and other viral infections: Efficacies and mechanisms. Pharmacol. Ther..

[88] Lui S.L., Zhu D., Cheng S.W., Ng F., Hui P.C., Yip T. (2015). Effects of astragalus membranaceus-based chinese medicine formulae on residual renal function in patients on peritoneal dialysis. Perit. Dial. Int..

[89] Lin Y.Q., Yu F., Chen H.J., Deng Y.R., Lin J., Xu Y., Zheng X., Zhang J.W., Liu J.F. (2024). Efficacy of astragalus combined with renin-angiotensin-aldosterone system blockers in the treatment of stage III diabetic nephropathy: A systematic review and meta-analysis. Ren. Fail..

[90] Teo W.Y., Chu S.W.F., Chow L.Y., Yeam C.T., Low L.L., Quah J.H.M. (2022). Role of Alternative Medical Systems in Adult Chronic Kidney Disease Patients: A Systematic Review of Literature. Cureus.

